# The Wire Rendezvous and Chasing Wire Technique in the Bidirectional Approach for the Percutaneous Coronary Intervention for Chronic Total Occlusion with a Single Guiding Catheter

**DOI:** 10.1155/2018/7162949

**Published:** 2018-10-30

**Authors:** Keisuke Nakabayashi, Daisuke Sunaga, Nobuhito Kaneko, Akihiro Matsui, Kazuhiko Tanaka, Hiroshi Ando, Minoru Shimizu

**Affiliations:** Kasukabe Chuo General Hospital, Heart Center, Saitama, Japan

## Abstract

A bidirectional approach for percutaneous coronary intervention for chronic total occlusion (CTO-PCI) using ipsilateral collaterals with a single guiding catheter limits procedural choices. The CTO of the left circumflex artery with ipsilateral collateral artery was treated by the bidirectional approach using a single guiding catheter. While the retrograde wire directly crossed the CTO lesion, the microcatheter could not pass the CTO lesion despite the conventional strategies. Therefore, we performed the wire rendezvous and chasing wire techniques. The wire rendezvous technique enables deeper retrograde guidewire progression, and the antegrade microcatheter can reach the CTO entry. The chasing wire technique enables the antegrade guidewire to pass the route made by the retrograde guidewire. These techniques might offer a possible solution for bidirectional CTO-PCI using a single guiding catheter. However, this technique should be considered as a last resort because of the risk of rapid reocclusion.

## 1. Introduction

Percutaneous coronary intervention (PCI) for chronic total occlusion (CTO) has improved not only patients' symptoms [1] and left ventricular function [2] but also long-term mortality [3, 4]. The advancement of techniques [5] and devices [6, 7] for CTO-PCI has contributed to this development. In addition, the procedural steps in current CTO-PCI have also been established [8]. Although the antegrade approaches, including the manipulation of stiff wires, knuckle wire technique, parallel wire technique, subintimal tracking and reentry (STAR) technique [9], contrast-guided STAR technique [10], or reentry devices [11, 12], are important, the retrograde approach is a particularly promising strategy in this era. The most commonly used collateral artery for the retrograde approach is the contralateral collateral artery, e.g., the right coronary artery (RCA) for the left coronary artery. However, some CTO lesions involve the ipsilateral collateral artery, such as the left anterior descending artery (LAD) to the left circumflex artery (LCX) or bridge collateral arteries. CTO-PCI with ipsilateral collateral channels is challenging [13]. A double guiding-catheter strategy, the so called “ping-pong” technique, could be one possible option, which leads to the smooth manipulation of the retrograde approach without interference from the antegrade system. This technique was reported for treating complications [14, 15] and CTO-PCI [16]. On the other hand, the single guiding-catheter strategy could be a less invasive option but limits the procedure choices because of the interferences in both systems. Herein, we present a novel strategy named the wire rendezvous and chasing wire technique. “Wire rendezvous” indicates full insertion of the retrograde wire into the antegrade microcatheter to advance the antegrade microcatheter tracking on the retrograde wire. “Chasing wire” indicates pushing the antegrade guidewire and pulling the retrograde guidewire simultaneously to let the antegrade guidewire track the route made by the retrograde guidewire before reocclusion. These techniques result in antegrade CTO crossing. These techniques could be options in the bidirectional approach for CTO-PCI using the single guiding catheter.

## 2. Case Presentation

**Figure 1:**
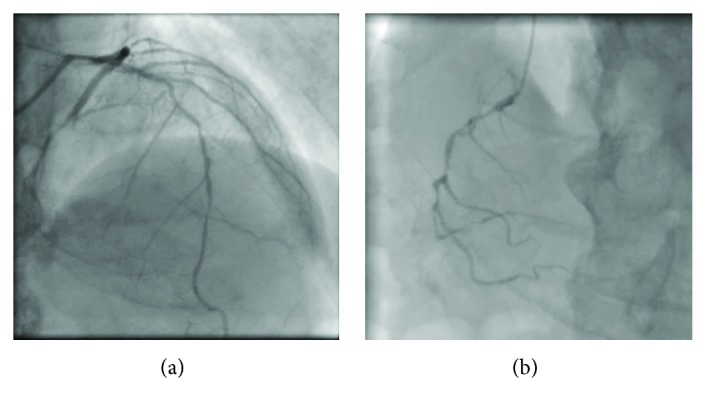
Angiography after the compensation of heart failure reveals severe stenosis with heavy calcification of the left anterior descending artery, chronic total occlusion of the left circumflex artery (a), and hypoplasty of the right coronary artery (b).

**Figure 2:**
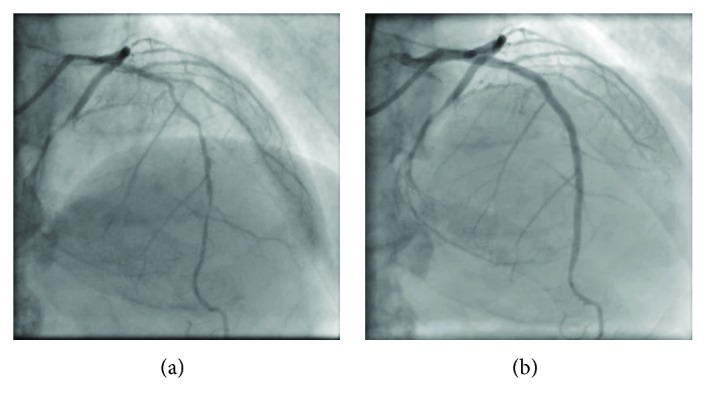
Angiographies before and after the percutaneous coronary intervention to the left anterior descending artery.

**Figure 3:**
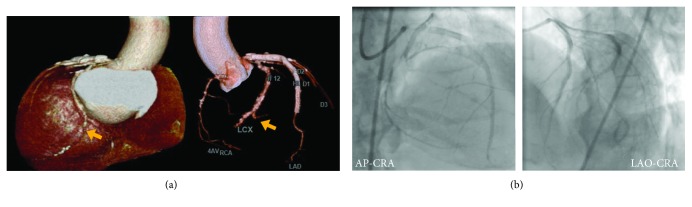
(a) Computed tomography after successful revascularization of the left anterior descending artery; the short and mildly calcified chronic total occlusion of the left circumflex artery; a stump presents after sending out the small branch (yellow arrows). (b) The middle left circumflex artery is occluded with Rentrop grade 2 collateral flow from the posterolateral branch channel and the apical channel. AP: antero-posterior; CRA: cranial; LAO: left anterior oblique.

A 78-year-old man with heart failure and low ejection fraction was referred to our institution. Electrocardiogram showed sinus rhythm, heart rate of 82 beats, and complete left branch bundle block. Laboratory data indicated 1.04 mg/dL of creatinine, 7.0% of HbA1c, and 268 pg/mL of brain natriuretic protein. Echocardiography showed an ejection fraction of 30%, left ventricle diastolic diameter of 60 mm, diffuse hypokinesis, and apical akinesis. Angiography after compensated heart failure revealed hypoplasty of the right coronary artery, severe stenosis with heavy calcification of the LAD, and CTO of the LCX (Figure 1). We first treated the LAD with standard stenting (Figure 2). Computed tomography after successful revascularization of LAD revealed a short and mildly calcified CTO; and a stump was revealed after sending out the small branch (Figure 3(a)). Thereafter, we tried to treat the LCX-CTO. The middle LCX was occluded with a Rentrop grade 2 collateral flow from the posterolateral branch channel and the apical channel (Figure 3(b), Videos 1–2). However, interventional collateral channels were unclear.

**Figure 4:**
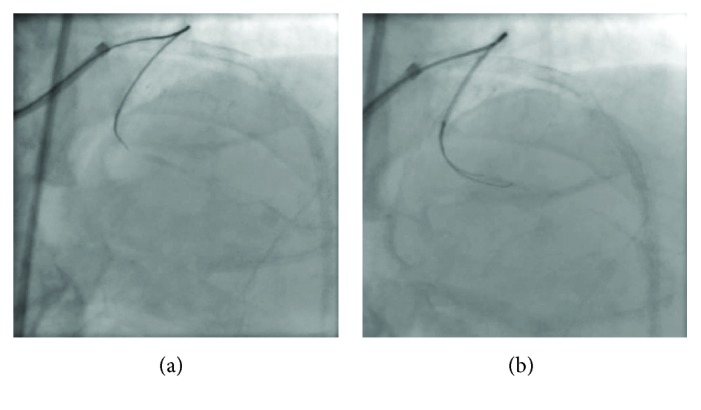
The antegrade approach with tapered wire (a) and stiff wires using the parallel wire technique (b) results in subintimal wiring.

**Figure 5:**
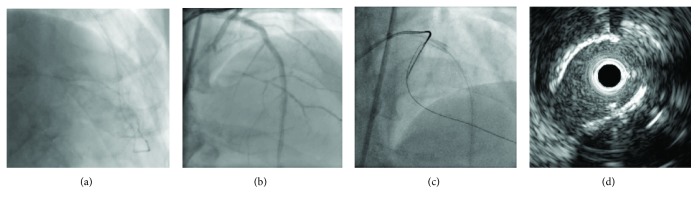
(a) Tip injection reveals the connection from the apical channel to the posterolateral branch. (b) The bidirectional angiography shows the short chronic total occlusion length. (c) The Gaia 2^nd^ directly crosses the chronic total occlusion lesion. (d) The intravascular ultrasound imaging confirms that the retrograde wire is in the true lumen.

**Figure 6:**
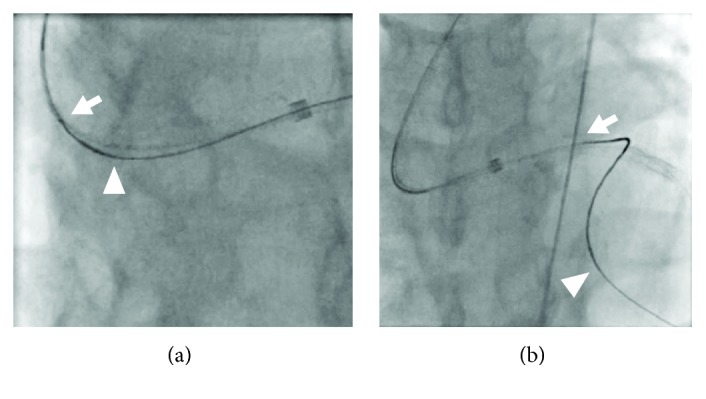
The wire rendezvous technique. (a) The insertion of the retrograde Gaia 2^nd^ into the antegrade Corsair Pro. (b) The antegrade Corsair Pro advance in the chronic total occlusion, tracking on the retrograde guidewire, but cannot pass the chronic total occlusion completely. The arrowheads indicate the tip of the Corsair Pro. The arrows indicate the tip of the retrograde Gaia 2^nd^.

**Figure 7:**
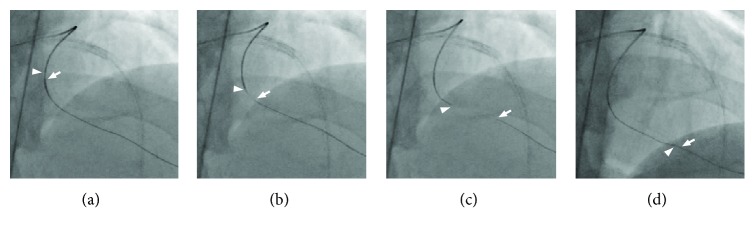
The chasing wire technique, pushing the antegrade guidewire and pulling the retrograde guidewire simultaneously. The arrowheads indicate the tip of the Corsair Pro. The arrows indicate the tip of the antegrade SION black. The arrows indicate the tip of the retrograde Gaia 2^nd^.

**Figure 8:**
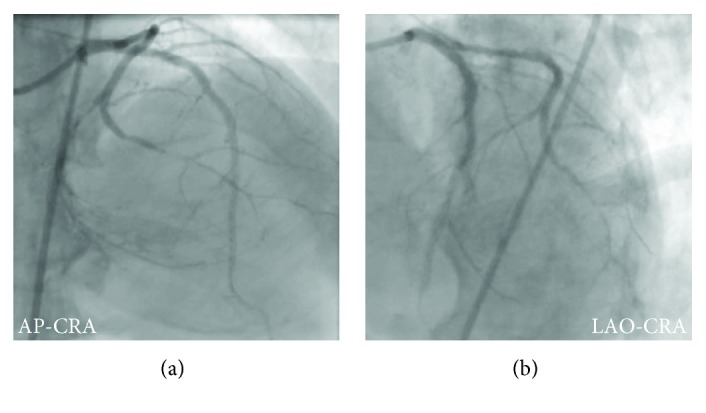
The final angiography. AP: antero-posterior; CRA: cranial; LAO: left anterior oblique.

We started PCI with the antegrade approach. We engaged an SPB 3.0, 8Fr (ASAHI Intecc, Nagoya, Japan) in the left coronary artery and progressed with the XT-R (ASAHI Intecc) supported by Corsair Pro (ASAHI Intecc) into the CTO stump. However, stiff wires and the parallel wire technique resulted in subintimal wiring (Figure 4). We chose to convert to the retrograde approach. Tip injection revealed that the apical channel was connected to the posterolateral branch (Figure 5(a)). The SUOH 03 (ASAHI Intecc) passed the channel and bidirectional angiography revealed the short CTO length (Figure 5(b)), and the Gaia 2^nd^ (ASAHI Intecc) directly crossed the CTO lesion (Figure 5(c)). Intravascular ultrasound imaging confirmed that the retrograde wire was in the true lumen (Figure 5(d)). However, a Mizuki (KANEKA MEDIX, Osaka, Japan) microcatheter could not pass the CTO lesion despite wire trapping by balloon catheter in the middle of the LCX. Even after the progression of the Gaia 2^nd^ directly into the guiding catheter, the microcatheter could not pass the CTO lesion. Attempts were made to instead use the new Caravel microcatheter (ASAHI Intecc), which is thinner and has a softer body; however, it could not pass the CTO lesion as well. Thus, we planned to catch the retrograde wire with a snaring catheter, but that did not work. Therefore, we performed the rendezvous technique that meant full insertion of the retrograde Gaia 2^nd^ into the antegrade Corsair Pro (Figure 6(a), Video 3). The antegrade Corsair Pro could advance into the CTO, tracking on the retrograde guidewire, but could not pass through the CTO completely (Figure 6(b)). Then, we tried the chasing wire technique, pushing the antegrade wire and pulling the retrograde wire simultaneously (Figure 7, Video 4). We chose the SION black (ASAHI Intecc) polymer jacket wire as an antegrade wire expecting smooth tracking along the route made by the retrograde guidewire before reocclusion. These techniques resulted in antegrade CTO crossing. We subsequently dilated the CTO with a small balloon and deployed the drug-eluting stent as usual (Figure 8, Videos 5–6).

## 3. Discussion

The CTO-PCI was developed along with novel techniques and devices [6, 7]. In particular, the bidirectional approach led to dramatic improvements in CTO-PCI success rates [5]. If interventionists established the bidirectional approach, some strategies, such as the direct retrograde guidewire crossing, kissing-wire technique, controlled antegrade and retrograde subintimal tracking (CART) technique [17, 18], or reverse CART technique [19], can be considered. After the retrograde guidewire has crossed the CTO lesion, the wire externalization can primarily be attempted and the retrograde microcatheter is required to reach the antegrade guiding catheter. Bidirectional approach for CTO-PCI with a single guiding catheter limits procedural choices in this situation. In the double guiding-catheter system, if the retrograde microcatheter could not track on the retrograde guidewire similar to our case, then (1) using a new microcatheter, (2) applying a guide-extension catheter, (3) performing wire trapping in the guiding catheter, or (4) catching the wire by snare catheter can be considered. Option 1 failed. Option 2, which utilized a small-sized catheter (e.g., 6Fr guide-extension catheter for 7Fr guiding catheter), was feasible, and the gap allowed the retrograde guidewire to progress in the antegrade guiding catheter; however, this was not considered at that time. In addition, options 3 and 4 are complicated when conducted via the bidirectional approach using the ipsilateral collaterals with the single guiding catheter. One solution could be the wire rendezvous technique and the chasing wire technique.

The wire rendezvous technique was first reported by Kim et al. [20]. They reported that the antegrade and retrograde microcatheters were aligned in the same guiding catheter, and the antegrade guidewire advanced into the retrograde microcatheter and reached the distal CTO lumen. The difference in the current case was that the retrograde microcatheter could not pass the CTO lesion. Therefore, we were required to perform the rendezvous technique inside the guiding catheter. There are recommendations regarding this approach. The rendezvous point should be at the greater curvature of the guiding catheter in the aortic sinus or aortic arch. Furthermore, inserting the guidewire near the tip of the microcatheter suppresses the microcatheter motion. In addition, several antegrade penetrations resulted in a large antegrade false lumen, which complicated securing the true lumen. Therefore, the antegrade microcatheter tracked on the retrograde guidewire, and the tip of that reached into the CTO entry, but not through the CTO lesion. This guaranteed that the antegrade guidewire was able to penetrate the true lumen from the CTO entry. Therefore, Corsair microcatheter could be suitable as the antegrade microcatheter.

To the best of our knowledge, the chasing wire technique has not been reported in the literature. The retrograde guidewire created a space with a width of 0.014 inches. Though this space might reocclude after wire retrieval, certain time lags could be expected. Therefore, we progressed the antegrade guidewire and pulled the retrograde guidewire simultaneously. The antegrade guidewire crossed the CTO through the space made by the retrograde guidewire. A polymer-coated wire, such as the SION black guidewire like our case, could be suitable as the antegrade wire because the surface of the CTO route might be narrow and rough. A tapered wire could also be appropriate in this situation, since the acute narrowing of the CTO route would occur after retrieving the retrograde guidewire.

Although the above techniques are technically feasible, they should be considered as last resort techniques because of the risk of rapid reocclusion. Since we easily succeeded in the direct retrograde guidewire crossing, recrossing seemed possible if needed. However, a very long CTO or a CTO requiring the CART technique can be riskier. In such cases, we should consider switching to the double guiding-catheter system by puncturing another access site.

## 4. Conclusion

Bidirectional approach for CTO-PCI using ipsilateral collaterals with single guiding catheter limits procedural choices. The wire rendezvous technique and the chasing wire technique might be possible solutions.

## Abbreviations

CTO: Chronic total occlusion
LAD: Left anterior descending artery
LCX: Left circumflex artery
PCI: Percutaneous coronary artery
RCA: Right coronary artery.

## Supplementary Materials

Supplementary 1 Video 1: the angiography before the percutaneous coronary intervention, antero-posterior view. The middle left circumflex artery is occluded with Rentrop grade 2 collateral flow from the posterolateral branch channel and the apical channel.

Supplementary 2 Video 2: the angiography before the percutaneous coronary intervention, left anterior oblique view.

Supplementary 3 Video 3: the wire rendezvous technique. The retrograde Gaia 2^nd^ advances into the antegrade Corsair Pro.

Supplementary 4 Video 4: the chasing wire technique; pushing the antegrade SION black wire and pulling the retrograde Gaia 2^nd^ wire simultaneously.

Supplementary 5 Video 5: the angiography after the percutaneous coronary intervention, antero-posterior view.

Supplementary 6 Video 6: the angiography after the percutaneous coronary intervention, left anterior oblique view.
